# The Microbiome of Potentially Malignant Oral Leukoplakia Exhibits Enrichment for *Fusobacterium, Leptotrichia, Campylobacter*, and *Rothia* Species

**DOI:** 10.3389/fmicb.2017.02391

**Published:** 2017-12-01

**Authors:** Abdrazak Amer, Sheila Galvin, Claire M. Healy, Gary P. Moran

**Affiliations:** ^1^Division of Oral Biosciences, School of Dental Science, Trinity College Dublin, Dublin Dental University Hospital, Dublin, Ireland; ^2^Division of Oral and Maxillofacial Surgery, Oral Medicine and Oral Pathology, School of Dental Science, Trinity College Dublin, Dublin Dental University Hospital, Dublin, Ireland

**Keywords:** microbiome, oral leukoplakia, oral cancer, *Fusobacteria*, Campylobacter, Rothia mucilaginosa

## Abstract

Oral leukoplakia presents as a white patch on the oral mucosa and is recognized as having significant malignant potential. Although colonization of these patches with *Candida albicans* is common, little is known about the bacterial microbiota of these patches. In the current study we analyzed the microbiome of oral leukoplakia in 36 patients compared to healthy mucosal tissue from the same patients and healthy control subjects to determine if specific microbial enrichments could be identified early in the malignant process that could play a role in the progression of the disease. This was carried out by sequence analysis of the V1–V2 region of the bacterial 16S rRNA gene using the Illumina MiSeq. Oral leukoplakia exhibited increased abundance of Fusobacteria and reduced levels of Firmicutes (Metastats *P* < 0.01). *Candida* colonization was also more prevalent in leukoplakia patients relative to healthy controls (*P* = 0.025). Bacterial colonization patterns on oral leukoplakia were highly variable and five distinct bacterial clusters were discerned. These clusters exhibited co-occurrence of *Fusobacterium, Leptotrichia*, and *Campylobacter* species (Pearson *P* < 0.01), which is strikingly similar to the microbial co-occurrence patterns observed on colorectal cancers (Warren et al., 2013). Increased abundance of the acetaldehydogenic microorganism *Rothia mucilaginosa* was also apparent on oral leukoplakias from lingual sites (*P* 0.0012). Severe dysplasia was associated with elevated levels of *Leptotrichia* spp. and *Campylobacter concisus* (*P* < 0.05). Oral leukoplakia exhibits an altered microbiota that has similarities to the microbiome of colorectal cancer.

## Introduction

Oral squamous cell carcinoma is the most common oral malignancy and is the eight most common cancer worldwide (Scully and Bagan, 2009). As with most cancers, failure to diagnose in the early stages of tumor development can have a dramatic impact upon long-term prognosis, with 5 year survival of late stage OSCC being less than 40% (Yanik et al., 2015). Early detection can be aided by the identification of potentially malignant precursors such as OLK, a condition that manifests as white, raised patches on the mucosal surface. The reported rate of transformation of OLK varies from 1 to >20% depending on the population and the length of follow up (Johnson et al., 2011; Yanik et al., 2015). Although smoking, alcohol and betel nut use are clearly associated with the development of OLK, factors that drive the malignant transformation of these lesions are poorly understood and it is difficult to accurately predict whether an OLK will resolve, persist or progress to OSCC (Liu et al., 2012). Malignant transformation of OLK is greater with increasing age, female gender, non-smoking, extent of OLK and may be affected by location, with some studies reporting that OLK on the lateral and ventral surfaces of the tongue and the floor of mouth having a higher rate of transformation (Silverman et al., 1984; Ho et al., 2012; Liu et al., 2012; Yanik et al., 2015). The degree of dysplasia is at present the most reliable indicator of the likelihood of transformation (Scully and Bagan, 2009; Liu et al., 2012).

A variety of hypotheses have been put forward to link microorganisms and their products with oral cancer (Perera et al., 2016). The production of known carcinogens such as *N*-nitroso compounds (Mills and Alexander, 1976) and acetaldehyde have been proposed as a way that microbes can induce OSCC (Marttila et al., 2013; Moritani et al., 2015). *Candida* colonization is often associated with OLK, which is referred to as “candidal leukoplakia” and is characterized by hyphal infiltration of the tissue (Abdulrahim et al., 2013; Alnuaimi et al., 2015). Some studies have associated *Candida* carriage with the degree of dysplasia (McCullough et al., 2002). Human papilloma virus is strongly associated with oro-pharyngeal cancer, but no clear role for it in the development of OSCC has been identified (Gillison et al., 2000).

More recently, microbiome studies have been carried out to identify changes in the bacterial microbiota in OSCC in the hope of identifying biomarkers of malignant transformation (Mager et al., 2005; Pushalkar et al., 2011; Hu et al., 2016). Nagy et al. (1998) carried out direct culture of OSCC lesions from 21 patients and identified high levels of *Porphyromonas, Prevotella*, and *Fusobacterium* species. Schmidt et al. (2014) analyzed the microbiome of OSCC in 27 patients by sequence analysis of the V4 region of bacterial DNA. OSCC patients exhibited reduced *Streptococcus* sp. and *Rothia* sp. but elevated levels of Bacteroidetes and Fusobacteria. Very recently, an enrichment for *F. nucleatum* and *P. aeruginosa* was identified in OSCC lesions from Yemeni patients (Al-hebshi et al., 2017). Although *F. nucleatum* is common in dental plaque and associated with oral infections, recent studies have also identified enrichment for *F. nucleatum* in colorectal cancer tissue (Castellarin et al., 2011; Kostic et al., 2011). Further studies have shown co-occurrence of *F. nucleatum, Leptotrichia* spp., and *Campylobacter* spp. on these tissues (Warren et al., 2013). *Fusobacterium* spp. have also been associated with cancers of the esophagus and were shown by PCR to be significantly enriched in esophageal cancer samples compared to normal esophageal mucosa (Yamamura et al., 2016). In the murine colon, it has been shown that *F. nucleatum* adheres to E-cadherin and Gal-GalNac expressed on tumors and this may also be the case in the oral cavity (Rubinstein et al., 2013; Abed et al., 2016). Molecular studies have shown that *F. nucleatum* can potentially promote tumor growth through activation of the Il-6-STAT3 axis and via activation of β-catenin signaling via the FadA adhesin (Rubinstein et al., 2013; Binder Gallimidi et al., 2015).

Although microbes such as *F. nucleatum* could potentially accelerate tumor development, most studies of the tumor microbiome have examined these lesions late in the malignant process. The current study was designed to examine the microbiome of potentially malignant OLK to determine if specific microbial enrichments could be identified early in the malignant process that could play a role in progression. Our study identifies a specific enrichment in Fusobacteria (both *Fusobacterium* spp. and *Leptotrichia* spp.) and *Campylobacter* spp. that bears similarity to recently identified enrichments on colorectal carcinoma.

## Materials and Methods

### Sample Collection

Ethical Approval for this study was granted by the Joint Hospitals’ Research Ethics Committee (Tallaght Hospital, Dublin). Following written informed patient consent, mucosal swabs were collected at the Dublin Dental University Hospital (DDUH) using Catch-all collection swabs (Epicentre, Madison, WI, United States). Patients presenting with OLK (*n* = 36, average age: 60.6) were swabbed at the site of the OLK and a contralateral normal site, where present (Supplementary Table S1). No normal sites were present in four patients and five patients presented with more than one OLK and were swabbed at both sites (Supplementary Table S1). Data on the degree of dysplasia identified on biopsy, the presence of dentures, smoking, alcohol consumption and oral hygiene measures were recorded. Patients having taken antibiotics or used topical steroids intra-orally in the past 6 months, were excluded along with patients with diabetes mellitus, Crohn’s disease, ulcerative colitis, current viral infection (cold/flu), or history of gastrointestinal malignancy. Healthy controls (*n* = 32, average age: 50.3) were subject to the same exclusion criteria and included 23 buccal swabs and 9 lingual (lateral border tongue) swabs.

### DNA Extraction

Swabs were resuspended in 300 μl of TSE buffer (10 mM Tris-HCl [pH 7.8], 1 mM EDTA, 100 mM NaCl) containing 500 U Ready-lyse lysozyme (Epicentre, Madison, WI, United States) and incubated at room temperature for 15 min. DNA extractions were carried out using the MasterPure DNA Purification Kit (Epicentre, Madison, WI, United States) using the manufacturer’s protocol with the addition of a bead disruption step, as follows: after Proteinase K treatment and before the addition of RNase, 0.25 glass beads (100 μM diameter) were added to the tube and the sample was disrupted in a Minibead beater (Biospec Products, Bartlesville, OK, United States) for 30 s. DNA pellets were resuspended in 35 μl TE buffer.

### DNA Sequencing

Amplification of the V1–V2 region of the 16S rRNA gene was carried using with the KAPA HiFi Hot start system (Kapa Biosystems) with the primers 27F-YM and 338R-R (27F-YM: 5′ TCGTCGGCAGCGTCAGATGTGTATAAGAGACAGAGTCAGTCTGTCAGAGTTTGATYMTGGCTCAG; 338R-R: 5′ GTCTCGTGGGCTCGGAGATGTGTATAAGAGACAGTATGGTAATTCATGCTGCCTCCCGTAGRAGT) (Frank et al., 2008; Marttila et al., 2013). The V1–V2 region was used as it has been shown that ∼90% of species in the Human Oral Microbiome Database (HOMD) can be correctly identified using this region (Diaz et al., 2014). Sample indexing was carried out with the Nextera XT Index Kit (Illumina) and library quantification and purification was carried out according to the Illumina protocol “16S Metagenomic Sequence Library Preparation” (Storey and Tibshirani, 2003; Abdulrahim et al., 2013; Illumina, 2013; Alnuaimi et al., 2015; Yanik et al., 2015). Size and integrity of indexed amplimers was determined using a Bioanalyzer (Agilent Technologies) and samples were normalized to 4 nM. Samples were combined to generate a pooled library, denatured and combined with PhiX control DNA (5%) and loaded at a concentration of 6 pM. Paired end sequencing was performed using the Illumina 600 cycle MiSeq reagent kit. All sequence data has been submitted to the NCBI sequence read archive (SRA), BioProject accession PRJNA394711.

### Sequence Analysis

Bacterial 16S rRNA sequences were analyzed using the Mothur pipeline (Schloss et al., 2009). Forward and reverse reads were aligned and filtered for quality and length (300–400 bp). Chimeric sequences were identified using Uchime (Edgar et al., 2011) and removed along with contaminating eukaryotic sequences and rare sequences (1–2 copies) prior to taxonomic classification. Sequences were classified in Mothur using the HOMD reference 16S rRNA gene set (V14.51). Operating taxonomic units (OTUs) were defined at a cutoff of 2% (i.e., 98% sequence identity), which our empirical analysis found was suitable for discrimination of the major oral taxa. OTU classifications from Mothur were supplemented by BLAST searches using consensus sequences to identify to the species level, where necessary. Mothur was used to calculate the Inverse Simpson index for each sample and to generate intra-sample rarefaction curves. For beta diversity analysis in Mothur, data were subsampled (normalized) to the smallest data set (3,709 sequences). Differences in community structure were inferred using distance matrices generated using the Bray–Curtis dissimilarity index calculated in Mothur (Schloss, 2008). Distance matrices were visualized using non-metric multidimensional scaling (NMDS) using the rgl package in R Studio.

### Statistical Methods

Statistical differences between microbial communities was identified using analysis of molecular variance (AMOVA). Taxonomic classifications that showed statistically significant differences in abundance in the study groups were identified using Metastats and LEfSe (White et al., 2009; Segata et al., 2011). Data on OTU abundance was formatted and normalized for LEfSe in Mothur and analyzed using the Galaxy server at https://huttenhower.sph.harvard.edu/galaxy/. *P*-values were corrected for multiple hypothesis testing using Bioconductor’s *q*-value package to estimate the false discovery rate (FDR) and associated *q*-values (Storey and Tibshirani, 2003). Taxa showing significant differences in abundance were reconfirmed using a Wilcoxon matched pairs test. Heatmaps were generated in R studio using Vegan to carry out hierarchical clustering on a matrix of Bray–Curtis dissimilarity values. Further statistical analysis was carried out using Prism (Graphpad Software, La Jolla, CA, United States).

### Quantitative PCR

To estimate carriage levels of *Candida* spp., we carried out quantitative Real-time PCR using primers targeting the *Candida* ITS2 rDNA region described by Kraneveld et al. (2012) (ITSF: 5′ CCTGTTTGAGCGTCRTTT; ITSR: 5′ TTCTCCGCTTATTGATAT). Amplification of the *Candida* ITS rDNA was performed with Fast Sybr Green master mix (Applied Biosystems) using the ABI 7500 Real-Time PCR System. *Candida* levels in each sample (CFU/ml) were extrapolated from a standard curve generated using DNA extracted from a serially diluted culture of *Candida albicans* SC5314 (10^8^ to 10 CFU/ml). CT values generated from the diluted *C. albicans* DNA samples were used to generate a standard curve using the Prism Software package and the number of *Candida* CFU/ml in all patient and control samples were determined by extrapolation from this standard curve. Data from the samples that were deemed *Candida* spp. positive contained DNA equivalent to at least 300 CFU/ml.

## Results

### Microbiome Composition

**FIGURE 1:**
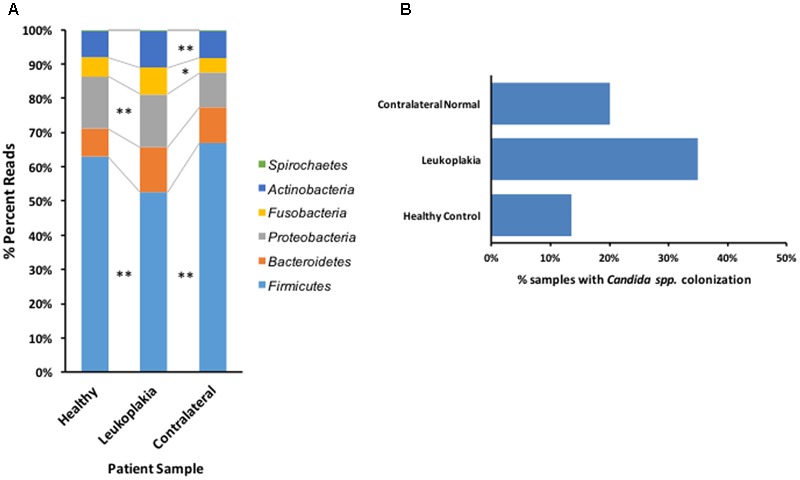
Overview of bacterial phyla distribution and *Candida* carriage patterns in patients with OLK and healthy controls. **(A)** Graph showing the abundance of the six most important phyla in healthy control subjects and sites of OLK and contralateral normal sites in patients. ^∗^ Indicates Metastats *P* < 0.05 and ^∗∗^*P* < 0.01. **(B)** Percentage of *Candida* positive samples recovered from patients (OLK and contralateral sites) and healthy controls, determined by qPCR.

Following sequence assembly and processing in Mothur, over 14 million sequences were included for analysis. These sequences were classified to 13 bacterial phyla representing 215 genera and 2,030 OTUs (clustered at 2% identity, Supplementary Table S2). Number of sequences in each sample are listed in Supplementary Table S3. Approximately 99% of sequences could be classified to the six most abundant phyla, Firmicutes, Bacteroidetes, Proteobacteria, Fusobacteria, Actinobacteria and Spirochaetes (**Figure 1**), with the remaining ∼1% belonging to the phyla *TM7, SR1*, Tenericutes, Chloroflexi and Synergistetes. Analysis of the abundance of these phyla using Metastats (White et al., 2009) revealed that leukoplakia samples had significantly lower levels of Firmicutes (*P* 0.0009) and higher levels of Proteobacteria (*P* 0.046) and Fusobacteria (*P* 0.0029) relative to contralateral normal sites. Levels of Firmicutes were also low relative to healthy control patients (*P* 0.008). Leukoplakia samples also exhibited higher levels of Bacteroidetes relative to healthy controls (*P* 0.0049), although these levels were not significantly higher than contralateral normal sites.

Levels of *Candida* colonization were assessed by quantitative PCR (**Figure 1B**). In total, 35% of samples from OLK sites showed significant colonization with *Candida* spp. (equivalent to at least 300 CFUs/ml). Colonization levels were lower at contralateral healthy mucosa from the same group of patients (20%) and in healthy control patients (13.5%). The majority (84%) of *Candida* spp. positive OLK were located at lingual sites (tongue, palate).

**FIGURE 2:**
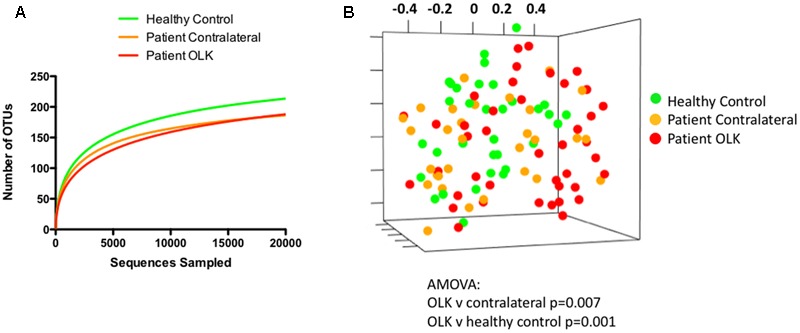
Analysis community structure in samples from OLK and healthy mucosa. **(A)** Rarefaction curve showing analysis of OTUs identified in samples from healthy controls (green) and patients (red = OLK tissue, orange = contralateral normal tissue). **(B)** Non-metric multidimensional scaling (NMDS) plots generated from a matrix of Bray–Curtis dissimilarity values using Mothur (Schloss et al., 2009). Separation of OLK communities (red) from contralateral (orange; *P* = 0.007) and healthy control communities (green; *P* = 0.001) was determined using analysis of molecular variance (AMOVA).

The species richness of the mucosal communities from healthy and OLK tissue were compared using species-accumulation (rarefaction) curves (**Figure 2A**). Analysis of the curves show that the number of OTUs begins to plateau above 5,000 sequences, indicating that the sampling effort is sufficient (only one sample of 104 yielded < 5,000 sequence reads, Supplementary Table S3). The curves also indicated that healthy control subjects exhibited greater species richness compared to samples recovered from OLK patients. Biodiversity of the communities was estimated using the Inverse Simpson index (Supplementary Table S3). Mean inverse Simpson values from patients and healthy controls were not significantly different.

To compare the structure of the bacterial communities from each sample, we determined the Bray–Curtis dissimilarity values for each patient sample using normalized data and generated a distance matrix from these data. Visualization of these data was carried out using NMDS (**Figure 2B**). Statistical differences in community structure were assessed using AMOVA. Mucosal health status appeared to influence population structure as bacterial communities from OLK samples exhibited significant separation from contralateral and healthy control mucosa in AMOVA tests (*P* < 0.007). However, smoking and the site of sampling (i.e., whether a buccal or lingual site) were both shown to have a greater influence on community structure (*P* < 0.001, Supplementary Figure S1).

### Characterization of Specific OTU Enrichments

The data set of all OTU abundances (Supplementary Table S4) was analyzed using LEfSe to identify significant enrichments in specific species or OTUs. Confirmation of these results was carried out using paired analysis of OLK and contralateral tissue for specific OTUs or species.

**FIGURE 3:**
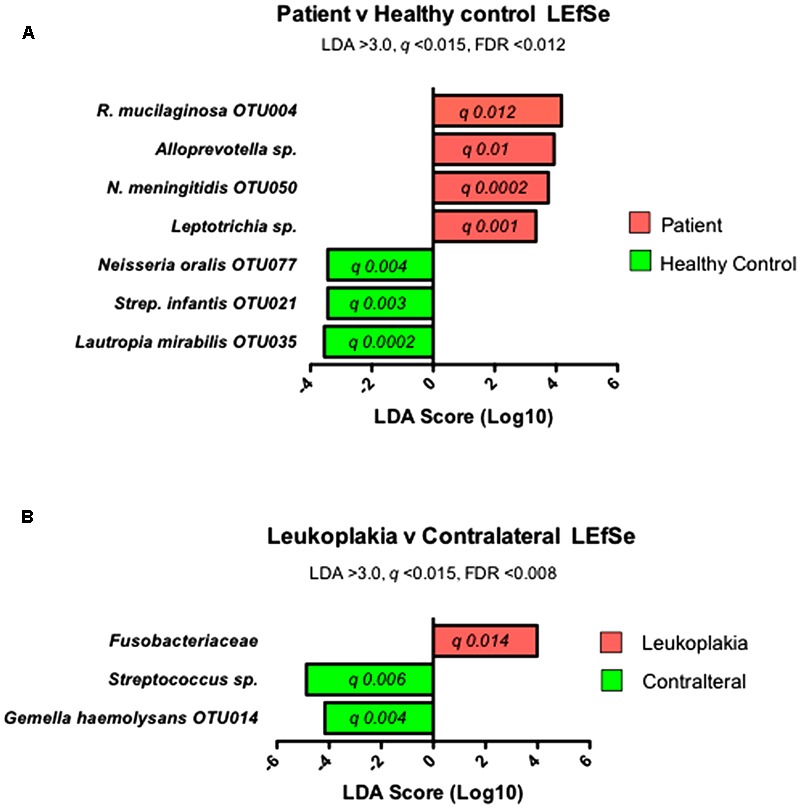
Results of LEfSe analysis to identify significantly enriched bacterial taxa in **(A)** communities from patients and healthy controls and **(B)** in communities from sites of OLK and contralateral tissue from patients.

Firstly, communities from OLK patients (combining both OLK and contralateral samples) were compared with communities from healthy controls. This analysis identified increased abundance of several taxa in OLK patients (*q* < 0.015, LDA > 3.0) including *Rothia mucilaginosa* (OTU004), *Alloprevotella* spp., *Neisseria meningitidis* (OTU050), and *Leptotrichia* spp. (**Figure 3A**). Next, we compared populations from OLK samples and contralateral normal sites from the same group of patients. This analysis identified increased abundance of Fusobacteriaceae on OLK (**Figure 3B**). Contralateral normal sites also exhibited increased abundance of *Streptococcus* spp. and *Gemella haemolysans* (OTU014).

**FIGURE 4:**
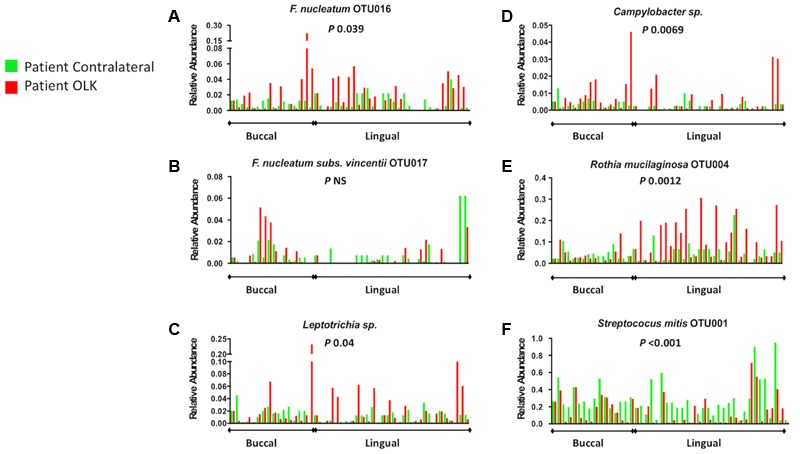
Relative abundance of selected taxonomic groups identified by LEfSe analysis. The abundance of each organism **(A–F)** in OLK and contralateral healthy communities from each patient is plotted side by side. No healthy tissues were available from four patients (Supplementary Table S1) and these are plotted versus the average values for control tissue. *P*-values refer to Wilcoxon matched pairs test results.

In order to confirm these enrichments in OLK communities, we carried out a paired analysis on matched leukoplakia and contralateral samples (**Figures 4A–F**). The Wilcoxon matched-pairs test confirmed that family Fusobacteriaceae (including *Fusobacterium* spp. and *Leptotrichia* spp.) were significantly greater in OLK communities relative to matched contralateral samples (*P* 0.024). This was significant for the largest fusobacterial taxon identified, *F. nucleatum* OTU016 (**Figure 4A**; *P* 0.039). Changes in abundance of *F. nucleatum* subsp. *vincentii* was site dependent with increased abundance in buccal OLKs, but conversely an increased abundance in lingual contralateral sites (**Figure 4B**). Increased abundance of *Leptotrichia* spp. (*P* 0.039), *Campylobacter* spp. (*P* 0.0069), and *R. mucilaginosa* (*P* 0.0012) was also shown in OLK communities relative to contralateral samples (**Figures 4C–E**). Conversely, *Streptococcus mitis* was significantly enriched in contralateral healthy samples (**Figure 4F**, *P* < 0.001).

Patient meta-data was also used to interrogate the abundance data using LEfSe (Supplementary Figure S2). Levels *F. nucleatum* OTU0016 and *Leptotrichia* sp. OTU044 were reduced in smokers but elevated in patients that were *Candida* carriers (*P* < 0.01). Consumption of alcohol (>1 unit/week) was associated with increased abundance of *Campylobacter* spp. (*P* 0.018). Use of mouthwash, age and sex of the patients did not significantly affect the level of the taxa under investigation.

**FIGURE 5:**
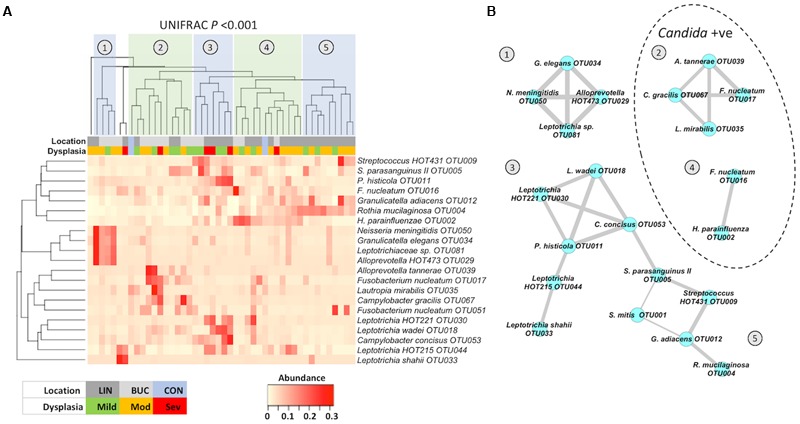
**(A)** Heatmap showing the abundance of taxa identified by LEfSe analysis. Heatmap and dendogram were generated in Vegan using a matrix of Bray–Curtis dissimilarity values. Separation of patient clusters marked 1 to 5 in the dendogram was highly significant (Unweighted UNIFRAC *P* < 0.001). The color-coded legend indicates whether each sample is from a buccal or lingual site and the degree of dysplasia following biopsy (mild, moderate, or severe). CON (light blue) correspond to the average values in healthy buccal (left) and healthy lingual (right) samples. **(B)** Co-occurrence map generated from Pearson correlation coefficients (*r*) generated in Prism (*P* < 0.01) and graphically presented using Cytoscape 3.2.1. Thickness of edges (connecting lines) are proportional to *r*-values (0.4–0.99). Clusters within the dotted line were identified in patients who were also colonized with *Candida* spp.

In order to determine whether any of these enriched bacterial taxa occurred together in specific communities on OLK, we generated a heatmap comparing the incidence of the 20 most abundant patient-enriched OTUs (Figure5A). Clustering of these profiles using Bray–Curtis dissimilarity values generated a dendogram with five major groups (labeled 1–5, **Figure 5A**) representing different enrichment patterns. Separation of these groups was statistically significant (UNIFRAC *P* > 0.001). Next, Pearson correlation coefficients were determined for each pair of OTUs and highly significant (*P* < 0.01) correlations were visualized using Cytoscape (**Figure 5B**). Five clusters representing the groups identified in **Figure 5A** were identified. Clusters (1) and (2) were found predominantly on buccal OLKs whereas Clusters (3), (4), and (5) were largely lingual. *S. mitis* OTU001 was negatively correlated with *S. parasanguinus* OTU005 (-0.444) and *G. adiacens* OTU012 (-0.375).

Patients in clusters (1), (3), and (5) were all *Candida* negative whereas 70% of patients in clusters 2 and 4 were *Candida* positive by qPCR. Severe dysplasia (*n* = 6) was recorded in biopsies from clusters (2), (3), and (4), with three cases (50%) of severe dysplasia in cluster (3). This cluster (3) exhibited enrichments for *Leptotrichia* spp. and *Campylobacter concisus* OTU0053. Comparison of the levels of these two organisms in all patients with mild, moderate, or severe dysplasia revealed that severe dysplasia was associated with elevated levels of *Leptotrichia* spp. and *C. concisus* OTU0053 (Supplementary Figure S3; Kruskal–Wallis *P* < 0.044).

## Discussion

Cancer is generally a multifactorial disease involving the accumulation of multiple genetic lesions. The involvement of the oral microbiome in the etiology or progression of OLK to OSCC has received relatively little attention. Most microbiome studies of OSCC have investigated the microbiome late in the malignant process and it is unclear whether these organisms adhere to the malignant tissue post-transformation or are drivers of the transformation itself (Nagy et al., 1998; Mager et al., 2005; Pushalkar et al., 2011; Schmidt et al., 2014; Hu et al., 2016). While some OSCCs arise *de novo*, others arise out of pre-existing lesions with malignant potential, the most common of which are OLKs. This study was designed to investigate differences in the bacterial community structure of OLK by comparing the microbiome of OLK samples with healthy mucosa from the same patient and control samples from healthy individuals. This initial investigation was designed to determine whether the mucosal microbiome was altered on OLK and whether this could be a potential driver of malignant transformation. Phyla level analysis of our data showed that significant changes in the abundance of three of the six most abundant oral phyla occurred in OLK compared to healthy mucosa from the same patients. We observed a decrease in the abundance of Firmicutes and an increase in the abundance of Fusobacteria and Actinobacteria. A decrease in Firmicutes and increase in Fusobacteria have been observed previously in OSCC (Schmidt et al., 2014). Our data indicate that these shifts occur early in the process of malignant transformation and may potentially play a role in its progression. As with previous studies, colonization with *Candida* spp. was also more prevalent on OLK compared to non-OLK sites.

Mucosal communities from OLK patients exhibited reduced species richness compared to healthy control subjects, but overall levels of biodiversity remained similar. Comparison of communities from healthy and diseased mucosa using the Bray–Curtis metric showed that community structure was significantly affected by the presence of leukoplakia (**Figure 2B**). However, our analysis indicated that the site (either buccal or lingual) and smoking had more significant impacts on community structure than whether the sample was recovered from OLK. In the current study smokers had significantly reduced levels of *Neisseria* sp. as recently reported (Wu et al., 2016). Smoking was also associated with reduced levels of *F. nucleatum* OTU016 and *Leptotrichia* sp. OTU044. The effects of smoking on the microbiome in OLK patients warrants further investigation as non-smokers are more likely to undergo malignant transformation than smokers (Silverman et al., 1984; Ho et al., 2012). Alcohol was also shown to affect the microbiome with alcohol consumption associated with enrichment for *Campylobacter* spp.

In general, OLK communities were found to be significantly enriched for Fusobacteriaceae, whereas contralateral sites exhibited higher levels of *Streptococcus* spp. and *Gemella* spp. Visualization of these data using a heatmap highlighted the heterogeneity in the patterns of enrichment. Five major clusters of OTUs could be identified (**Figure 5**), with clusters (1) and (2) generally associated with buccal OLK while clusters (3), (4), and (5) were largely lingual. Cluster (5) was exclusively lingual and exhibited enrichment for *R. mucilaginosa*, a normally abundant species in lingual communities. *R. mucilaginosa* typically accounted for ∼20% of sequences in enriched communities (compared to 6.7% of sequences on average in healthy patients). *Rothia* sp. are Gram positive species and are considered a part of the normal flora in the oropharynx and upper respiratory system (Kobayashi et al., 2012). One study examining the ability of oral microbes to produce acetaldehyde has shown that *R. mucilaginosa* is a potent producer of acetaldehyde (Moritani et al., 2015). ACH is a known carcinogen that can cause mutations in DNA and can cause sister chromatid exchanges and chromosomal defects in human cells. ACH has been found in the mouth after ethanol consumption (Homann et al., 1997). The high level of *R. mucilaginosa* identified in our study suggests that this organism could contribute to salivary ACH levels and studies are now ongoing to determine the ACH producing capacity of this species.

The remaining four clusters identified in **Figure 5B** all contained members of the Fusobacteriaceae, namely *F. nucleatum* and *Leptotrichia* spp. Cluster (2) included *F. nucleatum* subsp. *vincentii* (OUT017) and *Campylobacter gracilis*. Co-occurrence of Fusobacteria and *Campylobacter* sp. was also identified in the predominantly lingual cluster (3), which was enriched for *C. concisus* and *Leptotrichia* spp. The co-occurrence of *Fusobacteriaceae* with *Campylobacter* spp. observed in clusters (2) and (3) are strikingly similar to the co-occurrence patterns observed by Warren et al. (2013) on colorectal carcinomas. They identified a similar polymicrobial signature in an analysis of 130 colorectal carcinoma samples. Co-occurrence of *Fusobacterium, Leptotrichia*, and *Campylobacter* species were overrepresented on colorectal tumors and this enrichment was associated with specific changes in host gene expression, including increased pro-inflammatory IL-8 expression. As the natural niche for many of these organisms is the oral cavity, it is perhaps not surprising that we should also identify similar co-occurrence patterns on OLK, which suggests a strong predilection for these organisms to adhere to dysplastic tissue throughout the GI tract. Although only six of the leukoplakias analyzed here exhibited severe dysplasia, 50% of these were found in cluster (3) and analysis of all samples showed that severe dysplasia was significantly associated with elevated *Leptotrichia* spp. and *C. concisus* OTU053. Further studies are required to determine if Fusobacteria and *Campylobacter* spp. can exert synergy in accelerating tumor development, both in the colon and the oral cavity.

Examination of these data show that the species most enriched in OLK include *Fusobacterium, Leptotrichia, Campylobacter*, and *Rothia* species. *Candida* carriage was also prevalent on lingual OLKs and may influence the microbiota. Fusobacteria were the most consistent enrichment in all OLKs (both buccal and lingual). The most significant question posed by these data is whether these organisms influence the malignant transformation of OLK. Co-occurrence of Fusobacteria and *Campylobacter* spp. on OLK suggest that similar processes in malignant transformation may be at work in the oral cavity and in the colon, namely Fusobacterial activation of the Il-6-STAT3 axis and activation of β-catenin signaling. Work is ongoing to determine the oncogenic effects of these communities on oral cells and larger studies are underway to determine whether the presence of these communities can influence malignant transformation. If any of these communities can be implicated in malignant transformation, it may in the future be possible to identify those patients most at risk of developing OSCC and to perhaps use topical antibiotic therapy to prevent malignant transformation.

## Availability of Data and Material

All sequence data has been submitted to the NCBI sequence read archive (SRA), BioProject accession PRJNA394711.

## Ethics Statement

This study was carried out in accordance with the recommendations of the Trinity College Dublin “Good Research Practice” guidelines. All subjects gave written informed consent in accordance with the Declaration of Helsinki. The protocol was approved by the ‘Joint Hospitals’ Research Ethics Committee, Dublin.’

## Author Contributions

AA performed DNA extraction, PCR, DNA sequencing, data analysis, and manuscript preparation. SG was involved in data collection, study design, and manuscript preparation. CH was involved in study design, data collection, and manuscript preparation. GM was involved in study design, data analysis, and manuscript preparation.

## Conflict of Interest Statement

The authors declare that the research was conducted in the absence of any commercial or financial relationships that could be construed as a potential conflict of interest.

## Abbreviations

ACH: acetaldehyde
OLK: oral leukoplakia
OSCC: oral squamous cell carcinoma
