# Supplementary material for: The Microbiome of Potentially Malignant Oral Leukoplakia Exhibits Enrichment for Fusobacterium, Leptotrichia, Campylobacter, and Rothia Species
Source: Front Microbiol. 2017 Dec 1;8:2391. doi: 10.3389/fmicb.2017.02391 (PMC5717034; doi:10.3389/fmicb.2017.02391)
Supplement: Supplementary file 1 [file Table_1.DOCX]

**Table S1.** Characteristics of the patient population

| **Code** | **Age** | **OLK^a^** | **Normal^a^** | **Biopsy^b^** | **Smoker** | **Alcohol** | **Mouth Wash** | **Denture** |
| --- | --- | --- | --- | --- | --- | --- | --- | --- |
| **P1** | 65 | Rt LBT | Lt LBT | Mild dysplasia | NO | 1-2/wk | - | - |
| **P2** | 61 | Lt LBT | Rt LBT | Mild dysplasia | YES | 2-3u/mth | mouthwash 2x day | - |
| **P3** | 65 | Lt BM | Rt BM | Mod Dysplasia | YES | 1-2u/mth | - | - |
| **P4** | 85 | Lw LM | Up LM | Mod Dysplasia | NO | NO | - | Y |
| **P5** | 62 | Rt BM | Lt BM | Mild dysplasia | YES | NO | - | Y |
| **P6** | 58 | Rt FOM | Lt FOM | Sev dysplasia | YES | 10 u/wk | - | - |
| **P7** | 63 | Lt LBT | Rt BM | Mod dysplasia | YES | 8 u/wk | - | - |
| **P8(a)**  **(b)** | 40 | Rt BM Up LM | NA | Mild dysplasia  Mod dysplasia | NO | 1u/wk | - | - |
| **P9** | 55 | Lt BG | Rt BG | Mod dysplasia | YES | 1u/wk | mouthwash 1x day | Y |
| **P10** | 70 | Lt BM | Rt BM | Sev dysplasia | NO | 2-3u/mth | mouthwash 1 x day | Y |
| **P11** | 69 | D Ton | Rt BM | Mod dysplasia | NO | NO |  | Y |
| **P12** | 58 | Lt LA | Rt LA | Mod dysplasia | NO | 4-5u/wk | - | - |
| **P13** | 56 | Rt BM | Lt BM | Mod dysplasia | YES | 10u/wk | - | - |
| **P14** | 58 | Lt BM | Rt BM | Mod dysplasia | YES | 32 u/wk | - | - |
| **P15** | 79 | Lt HP | Rt HP | Mild dysplasia | NO | 6u/wk | - | Y |
| **P16** | 70 | Lt LBT | Rt LBT | Sev dysplasia | NO | 24u/wk | - | Y |
| **P17** | 66 | Rt FOM | Lt FOM | Mod dysplasia | YES | 20u/wk | - | - |
| **P18** | 65 | V Ton | Rt BM | Mod dysplasia | NO | NO | - | - |
| **P19 (a)**  **(b)** | 58 | SP  V Ton | NA | Mod dysplasia  Mod dysplasia | NO | 3-4 u/wk | mouthwash 1 x day | - |
| **P20** | 40 | Up Rt BG | Lw Rt BG | Mild dysplasia | YES | 8 u/wk |  | - |
| **P21** | 67 | FOM | D Ton | Sev dysplasia | YES | 1u/wk | - | Y |
| **P22** | 57 | Rt SP | Lt SP | Mild dysplasia | YES | NO | - | - |
| **P23** | 66 | V Ton | SP | Mod Dysplasia | YES | YES | - | Y |
| **p24** | 37 | Rt LBT | Lt LBT | Sev dysplasia | NO | NO | - | - |
| **p25** | 83 | Lt HP | Rt HP | Mod dysplasia | NO | NO | - | - |
| **p26** | 49 | Rt BM | Lt BM | Mod dysplasia | YES | NO | - | - |
| **p27 (a)**  **(b)** | 70 | V Ton BG | NA | Mild dysplasia  Mild dysplasia | NO | 5 /wk | mouthwash 1 x day | Y |
| **p28** | 40 | Lt LA | Rt BM | Mod dysplasia | YES | NO | - | - |
| **p29** | 58 | Rt LBT | Lt LBT | Mod dysplasia | YES | 5u/wk | - | - |
| **p30** | 57 | Lt SP | Lt BM | Mild Dysplasia | YES | 20u/wk | mouthwash 1 x day | - |
| **p31** | 62 | Lt BM | Rt BM | Sev dysplasia | NO | 6u/wk | mouthwash 1 x day | - |
| **p32 (a)**  **(b)** | 46 | Lt BM LA | Rt BM | Mod dysplasia  Mod dysplasia | YES | 20u/w | mouthwash 1 x day | - |
| **p33** | 76 | Lt LA | Rt LA | Mod dysplasia | NO | NO | - | Y |
| **p34** | 65 | Lt LBT | NA | Mod dysplasia | NO | 10u/wk | mouthwash 1 x day | - |
| **P35 (a)**  **(b)** | 63 | Lt LBT Rt BM | Rt BM | Mild dysplasia  Mild dysplasia | NO | 6-10u/wk | - | - |
| **P36** | 44 | Rt BM | Lt BM | Mod dysplasia | NO | 2-4 u/wk | - | - |
| **CM1** | 59 | Control | BM | Control | NO | 6-10 u/wk | - | - |
| **CM2** | 42 | Control | BM | Control | YES | 2-3 u/wk | - | - |
| **CM3** | 67 | Control | BM | Control | NO | 12-14 u/wk | mouthwash 1 x day | - |
| **CM5** | 55 | Control | BM | Control | NO | 5u/wk | - | - |
| **CM6** | 36 | Control | BM | Control | YES | 2-3/wk | - | - |
| **CM7** | 45 | Control | BM | Control | YES | 3-4 u/wk | - | - |
| **CM8** | 48 | Control | BM | Control | NO | 2u/wk | - | - |
| **CM9** | 42 | Control | BM | Control | NO | NO | - | - |
| **CM10** | 41 | Control | BM | Control | NO | 6u/wk | - | - |
| **CM11** | 66 | Control | BM | Control | NO | 7-9u/wk | - | - |
| **CM12** | 39 | Control | BM | Control | NO | NO | - | - |
| **CM13** | 41 | Control | BM | Control | NO | NO | - | - |
| **CM14** | 65 | Control | BM | Control | NO | 2 u/wk | - | - |
| **CM15** | 69 | Control | BM | Control | NO | 1 u/wk | mouthwash1 x day | - |
| **CM16** | 66 | Control | BM | Control | NO | 10 u/wk | - | - |
| **CM17** | 46 | Control | BM | Control | NO | NO | - | - |
| **CM18** | 60 | Control | BM | Control | YES | 40 u/wk | mouthwash1 x day | Y |
| **CM19** | 21 | Control | BM | Control | NO | NO | mouthwash1x day | - |
| **CM20** | 65 | Control | BM | Control | NO | 2-3 u/wk | mouthwash 1x day | Y |
| **CM21** | 68 | Control | BM | Control | NO | 2-3 u/wk | - |  |
| **CM22** | 53 | Control | BM | Control | NO | NO | - | - |
| **CM23** | 52 | Control | BM | Control | NO | 1 u/wk | - | - |
| **CM24** | 57 | Control | BM | Control | NO | 11 u/wk | mouthwash 1x day | - |
| **CT25** | 43 | Control | LBT | Control | NO | 2-3 u/wk | - | - |
| **CT26** | 66 | Control | LBT | Control | NO | 5 u/wk | - | - |
| **CT28** | 51 | Control | LBT | Control | YES | 2-3 u/wk | - | - |
| **CT29** | 58 | Control | LBT | Control | YES | NO | - | - |
| **CT30** | 45 | Control | LBT | Control | NO | NO | - | - |
| **CT31** | 43 | Control | LBT | Control | NO | NO | - | - |
| **CT32** | 30 | Control | LBT | Control | NO | 10 u/wk | - | - |
| **CT33** | 35 | Control | LBT | Control | NO | 4 u/wk | - | - |
| **CT34** | 35 | Control | LBT | Control | NO | 2 u/wk | - | - |

^a^Rt=right, Lt=Left, LBT=lateral border tongue, V Ton=ventral tongue, D Ton=Dorsum Tongue, BM=Buccal mucosa, HP=Hard palate, SP=Soft palate, LA=Lingual Alveolus, FOM=Floor of Mouth, BG=Buccal gingiva.

^b^Mod=Moderate, Sev=Severe
