# Supplementary figures and images for: The Microbiome of Potentially Malignant Oral Leukoplakia Exhibits Enrichment for Fusobacterium, Leptotrichia, Campylobacter, and Rothia Species
Source: Front Microbiol. 2017 Dec 1;8:2391. doi: 10.3389/fmicb.2017.02391 (PMC5717034; doi:10.3389/fmicb.2017.02391)

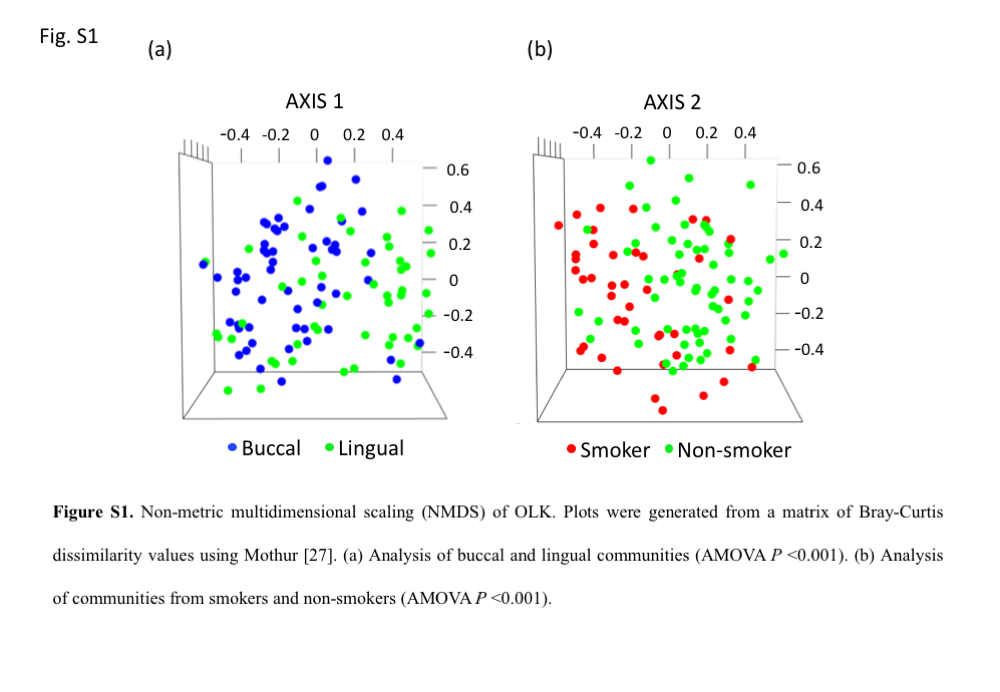

Supplement: Supplementary file 5 [file Image_1.TIFF]

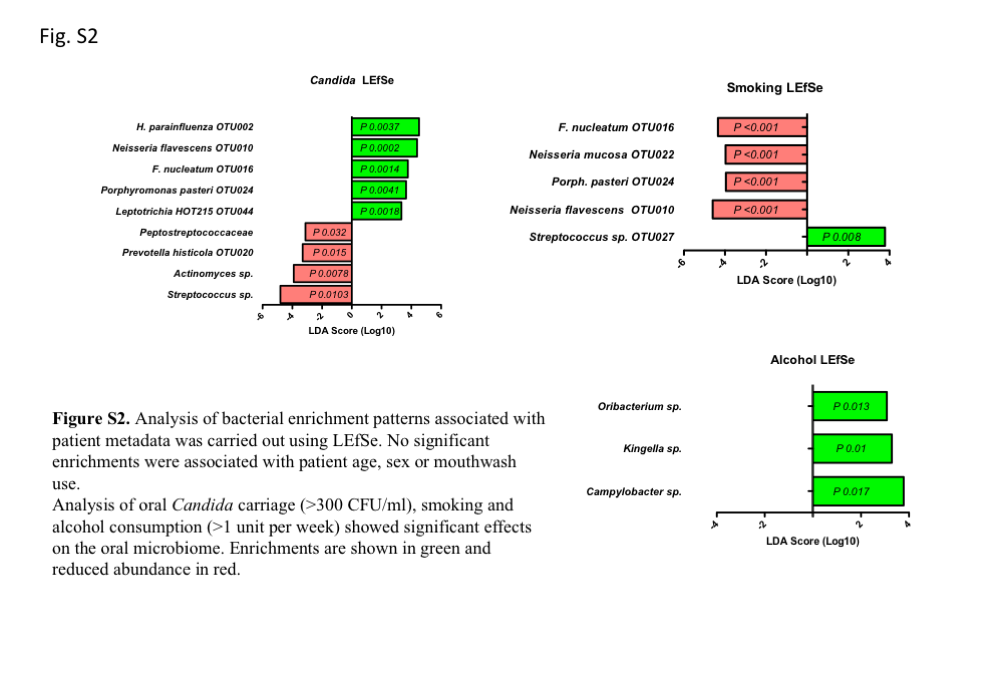

Supplement: Supplementary file 6 [file Image_2.TIFF]

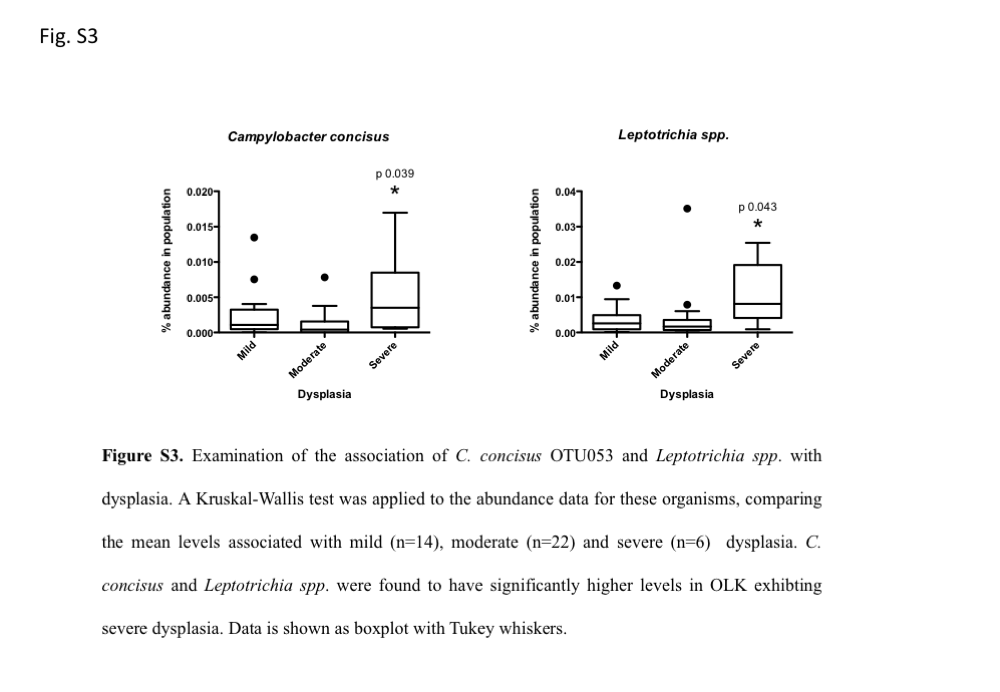

Supplement: Supplementary file 7 [file Image_3.TIFF]
